# Rapid Antidepressant Activity of Ethanol Extract of *Gardenia jasminoides* Ellis Is Associated with Upregulation of BDNF Expression in the Hippocampus

**DOI:** 10.1155/2015/761238

**Published:** 2015-03-24

**Authors:** Hailou Zhang, Wenda Xue, Runjie Wu, Tong Gong, Weiwei Tao, Xin Zhou, Jingjing Jiang, Ying Zhang, Nan Zhang, Yi Cui, Chang Chen, Gang Chen

**Affiliations:** ^1^Center for Translational Systems Biology and Neuroscience, School of Basic Biomedical Science, Nanjing University of Chinese Medicine, Nanjing 210023, China; ^2^Laboratory of Integrative Biomedicine of Brain Diseases, School of Basic Biomedical Science, Nanjing University of Chinese Medicine, Nanjing 210023, China; ^3^First Clinical Medical College, Nanjing University of Chinese Medicine, Nanjing 210023, China

## Abstract

Ethanol extract of Yueju pill, a Traditional Chinese Medicine herbal formula widely used to treat mood disorders, demonstrates rapid antidepressant effects similar to ketamine, likely via instant enhancement of brain-derived neurotrophic factor (BDNF) expression in the hippocampus. Here we investigated ethanol extracts of the constituent herbs of Yueju responsible for rapid antidepressant effects. Screening with tail suspension test in Kunming mice at 24 hours after a single administration of five individual constituent herbs of Yueju, we found that only* Gardenia jasminoides* Ellis (GJ) showed a significant effect. The antidepressant response started at 2 hours after GJ administration. Similar to Yueju and ketamine, a single administration of GJ significantly reduced the number of escape failures in the learned helplessness test. Furthermore, GJ decreased latency of food consumption in the novelty suppressed-feeding test. Additionally, starting from 2 hours and continuing for over 20 hours after GJ administration, BDNF expression in the hippocampus was upregulated, temporally linked with the antidepressant response. These findings suggest that GJ has rapid antidepressant effects, which are associated with the elevated expression of BDNF in the hippocampus. In Yueju formula, Yue represents GJ, as thus our study demonstrates the primary role of GJ in rapid antidepressant efficacy of Yueju.

## 1. Introduction

Depression, a widespread incapacitating psychiatric condition, imposes a substantial health threat to the society [1]. According to the World Health Organization prediction, depression will be the second leading contributor to common disease by the year 2020 [2]. The current first-line antidepressants are monoamine-based, which have some pronounced limitations. Most notably, it takes several weeks or longer for selective serotonin reuptake inhibitors (SSRIs), the most commonly prescribed antidepressants, to be fully effective. It is especially dangerous for individuals vulnerable to suicide due to the delayed onset of efficacy. Additionally, only about one-third of patients respond to the first medication of SSRIs prescribed [3]. Therefore, there is a pressing need to develop novel antidepressants in a fast-responding manner. Recent studies demonstrate that a single low dose of ketamine, a glutamate N-methyl-D-aspartic acid (NMDA) receptor antagonist, resulted in robust, rapid, and relatively sustained alleviation of depressive symptoms in depressive patients [4–6]. Ketamine also produces rapid antidepressant effects that last for several days in rodents [7, 8]. Since then, a handful of reagents have been identified to exhibit the nature of rapid antidepressant efficacy in preclinical studies [9].

In traditional Chinese medicine (TCM), a number of formulas have been created to alleviate symptoms of mood disorders. “Yueju” pill, formulated 800 years ago by a famous Chinese medicine Doctor Zhu Danxi, is frequently prescribed to treat depression and anxiety nowadays. Previous studies using animal models support the antidepressant effects of Yueju [10, 11]. We recently found that, similar to ketamine, ethanol extract of Yueju pill exhibited rapid antidepressant effects after a single administration, likely attributable to its instant upregulation of brain-derived neurotrophic factor (BDNF) expression in the hippocampus of mice [7, 12]. Similar to ketamine, Yueju may have a great clinical potential for treating patients with depression [13], particular to those who are resistant to conventional antidepressants.

Yueju consists of identical amount of five herbs:* Cyperus rotundus *L. (CR),* Ligusticum chuanxiong *Hort. (LC),* Gardenia jasminoides *Ellis (GJ),* Atractylodes lancea *(Thunb.) DC. (AL), and* Massa fermentata* (MF), most of which have shown antidepressant effects in various animal models. For example, ethanol extracts of AL, LC, or CR each reduced the immobility time in the tail suspension test (TST), a behavior despair paradigm to test antidepressant effect [11]. The ethanol extract of CR also reduced the immobility time in the forced swimming test and raised the concentration of 5-HT and dopamine in the prefrontal cortex [14, 15]. The antidepressant effect of ethanol extract of GJ was evidenced by its promotion of sucrose preference and the neuronal number in the hippocampus of mice [16]. However, all of these previous studies employed chronic drug treatment regimen, impossible to assess the rapid efficacy of an antidepressant. Therefore, the herb containing effective compounds crucial for rapid antidepressant efficacy of Yueju remains to be determined.

To address the issue, we first screened the constituent herbs of Yueju for rapid antidepressant effects using TST at 24 hours after a single administration. The identified herb was tested with additional behavioral paradigms for rapid antidepressant effects, including learned helplessness and novelty suppressed-feeding tests. Moreover, we monitored both antidepressant responses and BDNF expression in the hippocampus at various time points after a single administration of the effective herb to elucidate the underlying molecular mechanism.

## 2. Materials and Methods

### 2.1. Animals

Behavioral experiments were carried out using male Kunming mice (20–25 g), purchased from China Academy of Military Medical Sciences (Beijing). Mice, aged 6–8 weeks old, were habituated to animal facilities for 1 week before behavioral testing. The animals were maintained in standard laboratory conditions (temperature 22 ± 2°C and room humidity, 50 ± 10%) with a 12 : 12 h light/dark cycle. The animals were fed with standard diet and filtered water. The experimental procedures conformed to the Guide for the Care and Use of Laboratory Animals and were approved by the Institutional Animal Care and Use Committee at Nanjing University of Chinese Medicine.

### 2.2. Drugs

The medicinal plants used to prepare Yueju are* Cyperus rotundus *L. (CR),* Ligusticum chuanxiong *Hort. (LC),* Gardenia jasminoides *Ellis (GJ),* Atractylodes lancea *(Thunb.) DC. (AL), and* Massa fermentata *(MF). All of the medicinal plants were purchased from Nanjing Guoyi Clinical, Medicinal Material Department (Nanjing, China). All herbs were prepared as previously described [12]. Briefly, each constituent herb was powered, immersed in 95% ethanol for 24 hours with constant stir, and filtered. This procedure was repeated three times. The collected solvent was evaporated under reduced pressure and medium temperature (<55°C) to eliminate ethanol. The administered concentration was represented with the total amount of herb extract per kilogram of body weight. Ethanol extract of Yueju or a single constituent herb was dispersed in Tween 80 solution (0.5%, w/v in 0.9% saline). 0.5%, w/v Tween 80 in saline solution served as the vehicle control. The solutions of the herb preparation and vehicle were administered to mice via intragastric administration at a dosage of 0.1 mL/10 g (body weight). Ketamine HCl (Gutian Pharmaceuticals, China), dissolved in saline, was administered intraperitoneally and used as a positive control.

### 2.3. The Quality and Constitutes of GJ Ethanol Extract

Twelve batches of GJ were analyzed using HPLC fingerprint analysis. The high performance liquid chromatography (HPLC) analysis was performed on a Waters 2695 Alliance HPLC system (Waters Corp., Milford, MA, USA), equipped with a quaternary pump solvent management system, an online degasser, and an autosampler. The raw data were detected with 2998 DAD and processed with Empower Software. An Apollo C18 column (250 mm × 4.6 mm, 5 *μ*m) preceded by a Waters Symmetry Shield RP C18 guard column (20 mm × 3.9 mm, 5 *μ*m) was applied for all analyses. The injection volume was 10 *μ*L, and the column temperature was maintained at 30°C. The DAD detector was set at 265 nm for acquiring chromatograms. The mobile phase was composed of A (methanol) and B (0.1% aqueous acetate acid, v/v) with a gradient elution: 0–15 min, 10–20% A, 15–25 min, 20–55% A, 25–40 min, 55–90% A, 40–45 min, and 90–100% A. The flow rate of the mobile phase was 1.0 mL/min. HPLC-DAD chromatographic data of the 11 tested samples were submitted for analysis by using the professional software “Similarity Evaluation System for Chromatographic Fingerprint of TCM” (Version 2004 A) to extract the mean chromatogram and the similarities.

### 2.4. Tail Suspension Test

In acoustic and visual isolated chambers, a single mouse was suspended in 50 cm above the floor, with a tape placed at about 1 cm of the tail. Activities of the animals were videotaped. The computer calculated the total duration of immobility during the last 4 min in a 6 min testing time [17]. To reduce the use of animals, the brains were harvested immediately after the test for Western blotting analysis in vehicle, GJ, and ketamine administrated mice. A pilot analysis indicated that this behavior testing did not influence thereafter BDNF protein expressions.

### 2.5. Learned Helplessness Test

The procedures for learned helplessness were followed as reported previously, with minor modifications [18, 19]. Learned helplessness experiments were performed in soundproofed two-way shuttle boxes (40 × 10 × 13 cm), with walls made of clear Plexiglas. The chamber was divided into two identical compartments. For the induction of helplessness, mice received 120 inescapable shocks (18~44 s, average 30 s; 0.45 mA for 15 s) once daily for 2 consecutive training days. Two hours after termination of the helplessness induction, mice were treated with GJ, Yueju, ketamine, or vehicle. Mice were tested for helplessness 24 hours after the drug treatment. For testing of learned helplessness response, animals were subjected to 30 avoidance trials (18~44 s, average 30 s; 0.45 mA for 3 s).

### 2.6. Open Field Test

Open field test was used to assess the locomotory as well as the exploratory behavior in open area. In the test, spontaneous locomotor activity was measured in a square arena (40 × 40 × 15 cm) for monitoring horizontal activity, namely, total distance traveled. Mice were tested in a well-illuminated (~300 lux) transparent acrylic cage for 5 min. Activity of mice in the two compartments, near the bulkhead and central regions, was tracked. Distance (cm) and time spent in the central zone were analyzed. Test device was thoroughly cleaned before each animal using 75% ethanol.

### 2.7. Novelty Suppressed-Feeding Test

Mice were food-deprived for 24 h and then placed into a 40 × 40 cm open field. A single pellet of the mouse's normal food chow was placed in the center of the open field arena. Each animal was placed in a corner of the arena at the beginning and allowed to explore for up to 10 min. The trial ended when the mouse chewed a part of the chow. The amount of food consumed in the home cage was taken as the weight of chow consumed, as a control measure for appetite. The latency to begin eating, defined as active chewing of the pellet, was recorded.

### 2.8. Western Blotting

The whole hippocampus (ventral and dorsal) were rapidly dissected, frozen, and lysed in buffer containing protease inhibitors and phosphatase inhibitors. Total protein concentration was quantified by Bradford analysis. Protein concentration was determined colorimetrically by BCA assay (Pierce, Rockford, IL, USA). Protein lysates were separated by 12% SDS-PAGE electrophoresis and were transferred onto polyvinylidene difluoride (PVDF) membranes. BDNF quantification was carried out by SDS-polyacrylamide gel electrophoresis. Primary antibodies for BDNF (Santa Cruz Biotechnology) and *β*-tubulin (Cell Signaling) were used at dilutions of 1 : 200 and 1 : 5,000, and anti-rabbit secondary antibodies were used at 1 : 2,000 and 1 : 5,000, respectively. The blots were visualized using the Super Signal West Pico Chemiluminescent Substrate (Thermo Fisher Scientific Inc.). The amount of BDNF was normalized to *β*-tubulin bands. All experiments were performed 3 times.

### 2.9. Statistical Analysis

Data presented are mean ± SEM. Statistical evaluation was performed by one-way analysis of variance (ANOVA) with least significant difference (LSD) multiple comparison test. A value of *P* < 0.05 was considered statistically significant.

## 3. Results

### 3.1. Fingerprint of Ethanol Extract of GJ

**Figure 1:**
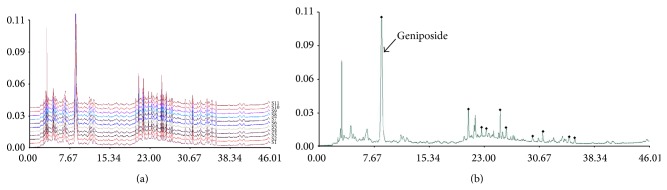
Fingerprints of GJ. (a) The chromatographic fingerprints of twelve samples of GJ (S1–S12) and (b) the mean chromatographic fingerprint developed with the software “Similarity Evaluation System for Chromatographic Fingerprint of TCM.” The 10 common peaks are labeled.

Twelve samples of GJ were used to develop the standard fingerprints (Figure 1(a)). The mean chromatographic fingerprint obtained from the software “Similarity Evaluation System for Chromatographic Fingerprint of TCM” was shown in Figure 1. Peaks presented in all 12 samples were defined as “common peaks.” As a result, 10 characteristic peaks shown in the fingerprint chromatogram were assigned as common peaks (Figure 1(b)). The similarity of each chromatogram to the mean chromatogram was greater than 0.9, indicating that the quality of the 12 batches was very similar and suitable.

### 3.2. GJ Showed an Antidepressant Effect in TST at 24 Hours after a Single Administration

**Figure 2:**
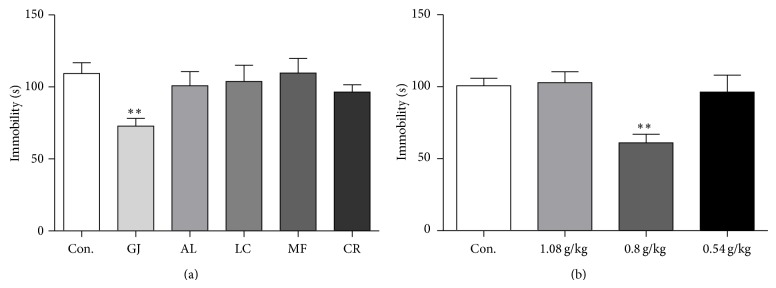
Identification of the herb primary for rapid antidepressant effect of Yueju at 24 hours after a single administration. Immobility time was measured for the last 4 minutes during the 6-minute testing time of TST. (a) Effects of individual constituent herbs of Yueju at 0.8 g/kg. Groups: Con. (control), GJ, AL, LC, MF, and CR. One-way analysis of variance (ANOVA),* F*_(5,59)_ = 2.58, *P* < 0.05, and *n* = 10/group, ^**^ *P* < 0.01 versus control (LSD post hoc tests). (b) Dose-effect relationship of GJ. ANOVA,* F*_(3,47)_ = 5.97, *P* < 0.05, and *n* = 12/group, ^**^ *P* < 0.01 versus control (LSD post hoc tests).

Individual ethanol extracts of constituent herbs of Yueju were screened for rapid antidepressant effect, using TST paradigm carried out 24 hours after a single administration, a time point when Yueju and ketamine demonstrate rapid antidepressant efficacy by decreasing immobility time in this paradigm and reversed deficits in learned helplessness paradigm [8, 12, 18]. At the dosage of 0.54 g/kg, equivalent to the dosage of each constituent herb in Yueju, there was no effect of treatment (see Supplemental Figure S1 in the Supplementary Material available online at http://dx.doi.org/10.1155/2015/761238). However, at dosage of 0.8 g/kg, there was a significant effect of treatment (Figure 2(a), ANOVA,* F*_(5,59)_ = 2.58, *P* < 0.05, and *n* = 10), with GJ the only effective herb (*P* < 0.01, LSD post hoc tests). The dose-effect relationship of GJ was confirmed in an independent experiment, with higher or lower dosage ineffective (Figure 2(b), ANOVA,* F*_(3,47)_ = 5.97, *P* < 0.05, and *n* = 12).

### 3.3. The Antidepressant Effect of GJ Started at 2 Hours after an Administration

**Figure 3:**
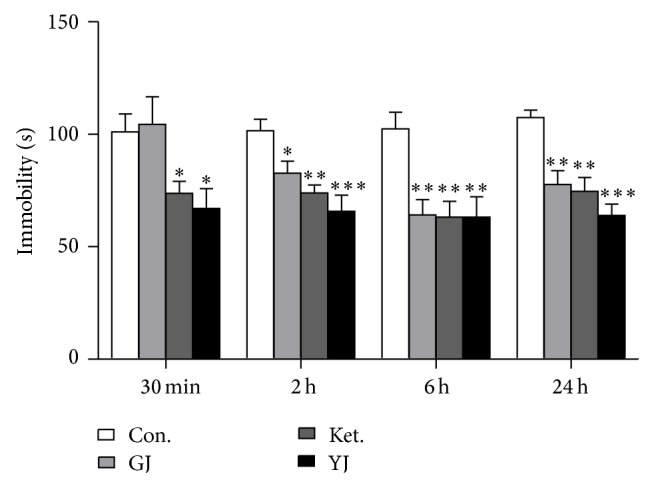
Antidepressant effects of GJ using the tail suspension test at different times after a single administration. Immobility time was measured for the last 4 minutes during the 6-minute testing time at 30 min, 2 h, 6 h, and 24 h after a single GJ administration. Groups: Con. (control), GJ (0.8 g/kg), Ket. (ketamine, 30 mg/kg), and YJ (Yueju, 2.7 g/kg). One-way analysis of variance (ANOVA), 30 min:* F*_(3,31)_ = 4.485, *P* = 0.011, and *n* = 8/group; 2 h:* F*_(3,31)_ = 8.021, *P* = 0.001, and *n* = 8/group; 6 h:* F*_(3,31)_ = 6.545, *P* = 0.002, and *n* = 8/group; 24 h:* F*_(3,39)_ = 8.942, *P* = 0.0001, and *n* = 10/group. ^*^ *P* < 0.05, ^**^ *P* < 0.01, and ^***^ *P* < 0.001, versus control. Data represent mean ± SEM.

The antidepressant effects of GJ were further examined over time after a single administration, using Yueju and ketamine as positive controls. To avoid the effects of repeated testing, only independent animals were used. There was a significant effect of treatment at different time points (ANOVA, 30 min,* F*_(3,31)_ = 4.485, *P* = 0.011; 2 h,* F*_(3,31)_ = 8.021, *P* = 0.001; 6 h,* F*_(3,31)_ = 6.545, *P* = 0.002; 24 h,* F*_(3,39)_ = 8.942, *P* = 0.0001). As expected, from 30 min to 24 h after a single administration, both Yueju (2.7 g/kg) and ketamine (30 mg/kg) showed significant antidepressant effects (*P* < 0.05, versus control). GJ showed significant antidepressant effects at 2, 6, and 24 h after a single administration (2 h, *P* = 0.02; 6 h, *P* = 0.001; 24 h, *P* = 0.003, versus control, resp.). However, GJ did not have an effect at 30 min after administration (*P* = 0.792) (Figure 3).

### 3.4. GJ Rapidly Alleviated Deficits in Learned Helplessness

**Figure 4:**
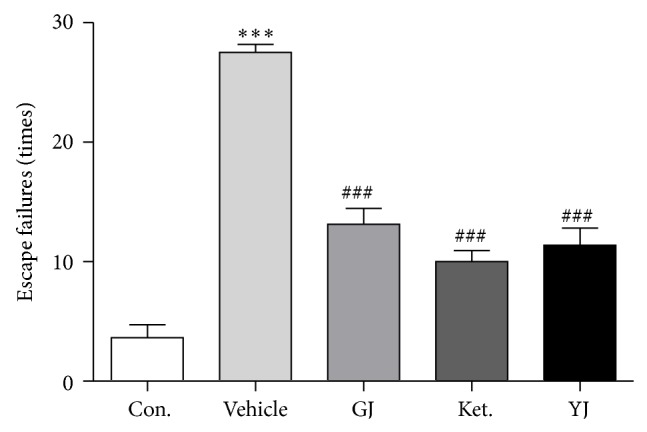
Rapid antidepressant effects of GJ in the learned helplessness paradigm. Learned helplessness was quantified as number of escape failures in an active avoidance test. Mice received inescapable shock training for two days prior to a 30-trial active avoidance test. ANOVA,* F*_(4,39)_ = 60.793, *P* < 0.05, and *n* = 8/group, ^***^ *P* < 0.001 compared to control, ^###^ *P* < 0.001 compared to vehicle, LSD post hoc tests.

Learned helplessness behavior was tested at 24 h after a single administration of GJ. There was a significant effect of treatment (Figure 4, ANOVA,* F*_(4,39)_ = 60.793). Compared to vehicle, GJ significantly reduced the number of escape failures (*P* < 0.001), an effect that was also observed in mice administrated with Yueju (*P* < 0.001) or ketamine (*P* < 0.001).

### 3.5. GJ Rapidly Reduced Novelty Suppressed-Feeding Response

**Figure 5:**
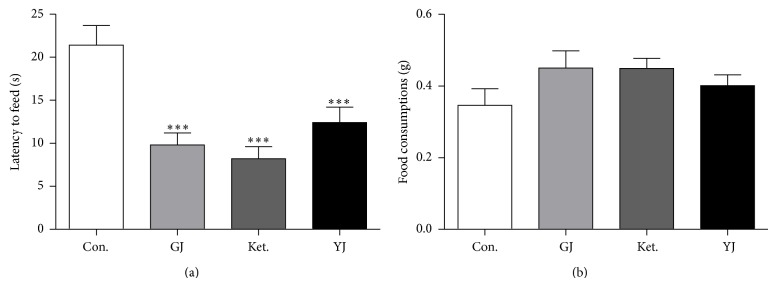
Effects of GJ on the novelty suppressed-feeding behaviors. (a) GJ, ketamine, and Yueju all significantly decreased the time of latency to eat during 10 min test,* F*_(3,39)_ = 11.223, ^***^ *P* < 0.001, and *n* = 10/group. (b) There was a trend for the effect on the total amount of food consumed,* F*_(3,39)_ = 1.598, *P* = 0.207, and *n* = 10/group. GJ (*P* = 0.067) and ketamine (*P* = 0.070) with post hoc tests.

In the novelty suppressed-feeding test, a single administration at 24 hours, the latency to eat decreased significantly in mice treated with GJ (*P* < 0.001), ketamine (*P* < 0.001), or Yueju (*P* < 0.001). (Figure 5(a), ANOVA,* F*_(3,39)_ = 11.223, *P* < 0.05). Additionally, GJ tended to increase the amount of food consumed (Figure 5(b), ANOVA,* F*_(3,39)_ = 1.598, *P* = 0.207).

### 3.6. GJ Did Not Influence Behaviors in the Open Field Test

**Figure 6:**
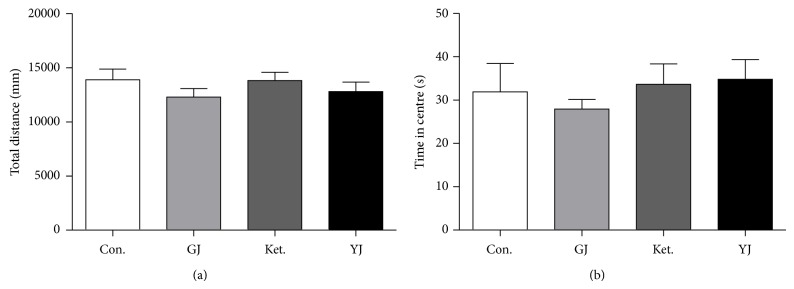
Effects of GJ on the open field test. Mice were injected with saline, GJ (0.8 g/kg), Ket. (30 mg/kg), or YJ (2.7 g/kg) and tested at 24 h after injection. Values represent mean ± SEM. (a) T distance traveled during a 5 min open field testing time, ANOVA,* F*_(3,39)_ = 0.826, *P* = 0.488, and *n* = 10/group. (b) Time spent on the center part during a 5 min open field testing time. ANOVA,* F*_(3,39)_ = 0.403, *P* = 0.752, and *n* = 10/group.

The effective antidepressant doses of GJ, ketamine, or Yueju after drug administration did not change either locomotor activity (total distance) (Figure 6(a), ANOVA,* F*_(3,39)_ = 0.826, *P* = 0.488) or time spent on the center part of open field (Figure 6(b), ANOVA,* F*_(3,39)_ = 0.403, *P* = 0.752), a measurement of the level of anxiety.

### 3.7. Time Course of BDNF Expression in the Hippocampus after a Single GJ Administration

**Figure 7:**
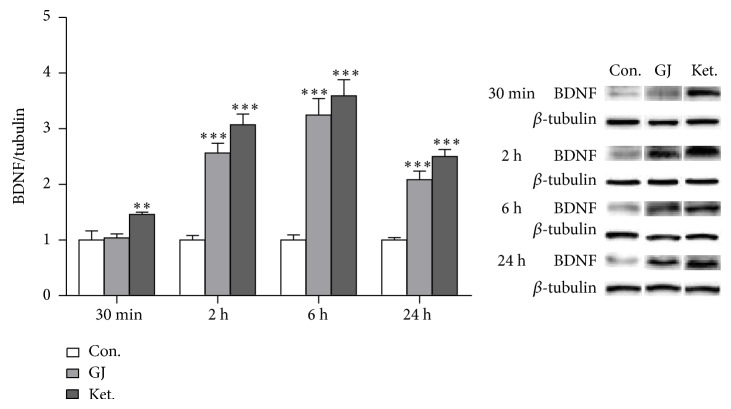
BDNF expressions in the hippocampus over time after a single administration of GJ. BDNF expression significantly increased from 2 h to 24 h after a single GJ administration and from 30 min to 24 h after a single ketamine administration. *n* = 6/group. ^***^ *P* < 0.001, LSD post hoc tests.

The BDNF protein level was found to be associated with rapid antidepressant effect of ketamine and Yueju previously in preclinical models [7, 12]. To understand the molecular mechanism underlying antidepressant effects of GJ, we examined the expression of BDNF in the hippocampus at different time points after a single administration of GJ or ketamine. There was a significant treatment effect at all-time points examined, including 30 min (ANOVA,* F*_(2,17)_ = 5.831, *P* < 0.05), 2 h (*F*_(2,17)_ = 48.0746, *P* < 0.05), 6 h (*F*_(2,17)_ = 32.875, *P* < 0.05), and 24 h (*F*_(2,17)_ = 43.339, *P* < 0.05) after a single administration. GJ significantly increased the BDNF expression at 2 h, 6 h, and 24 h after an administration (*P* < 0.001 at each time point). However, there was no effect of GJ at 30 min, in contrast to the increased BDNF expression by ketamine (*P* < 0.01) or Yueju [12] (Figure 7).

### 3.8. Geniposide Did Not Show Antidepressant Effect at 24 Hours after a Single Administration

**Figure 8:**
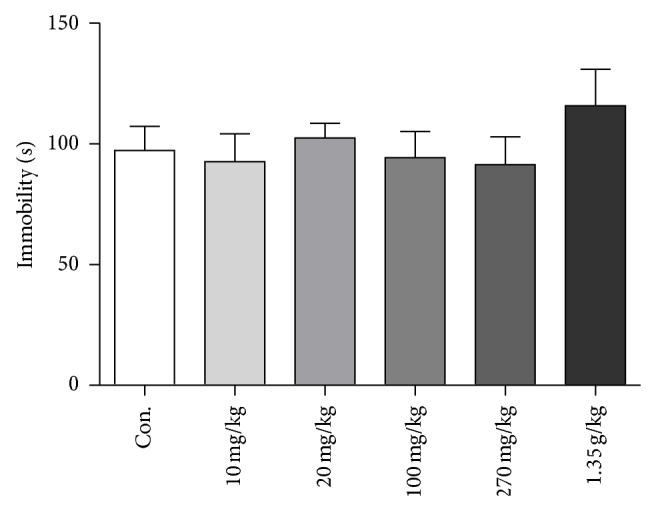
Effects of geniposide on the tail suspension test in mice at 24 h after a single administration. Immobility time was measured for the last 4 minutes during the 6-minute testing time. Geniposide concentration group included 10 mg/kg, 20 mg/kg, 100 mg/kg, 270 mg/kg, and 1.35 g/kg. One-way analysis of variance (ANOVA),* F*_(5,71)_ = 0.662, *P* = 0.654, and *n* = 12. Data represent mean ± SEM.

Geniposide is a hallmark compound enriched in GJ. A number of studies suggest its antidepressant efficacy, which may contribute to rapid antidepressant of GJ [20, 21]. We found there was no significant effect on the immobility time in TST at 24 h after a single administration of variable concentration of geniposide (ANOVA,* F*_(5,71)_ = 0.662, *P* = 0.654, and *n* = 12) (Figure 8).

## 4. Discussion

Our previous study showed that Yueju, an herb formula invented for treatment of mood disorders, has rapid antidepressant effects. To identify the herb primary for Yueju's function, we first assessed the antidepressant effect using TST at 24 h after a single administration of each of constituent herbs of Yueju and found that only GJ showed effects similar to Yueju. Furthermore, GJ showed rapid antidepressant responses in the learned helplessness paradigm and NSF paradigm. Interestingly, the increase of BDNF expression at various times after GJ administration paralleled with the time course of antidepressant response, from 2 h to 24 h after a single administration. These findings suggest that GJ has rapid antidepressant effects, which are associated with acutely increased expression of BDNF in the hippocampus.

The present study showed that GJ is able to quickly act as an antidepressant, using various behavioral paradigms. A single administration of GJ and Yueju resulted in improvement in the novelty suppressed-feeding behavior, which requires repeated administrations of monoamine-based antidepressants in the rodent model [8], supporting the fast action nature of GJ and Yueju. Similar to ketamine and Yueju, a single administration of GJ also significantly alleviated the behavioral deficits in learned helplessness paradigm, which may also indicate a rapid antidepressant potent [8]. Tail suspension test is one of the most widely used models for assessing the antidepressant effects [22]. We found that the GJ was the only constituent herb of Yueju that showed antidepressant effects sustained for 1 day after a single administration. A previous study using chronic administration of much higher dose of GJ did not detect the antidepressant effect in TST [11], in agreement with our findings that high dose of GJ was ineffective. The restricted dose range was observed in Yueju for rapid antidepressant effects [12]. Indeed, high dose of ketamine also failed to show rapid antidepressant effects and only appropriate dose of ketamine can activate mammalian target of rapamycin (mTOR) signaling leading to upregulation of synaptic plasticity for antidepressant responses [8]. The mechanism may also be shared for Yueju or GJ. Taken together, these findings support that GJ has rapid antidepressant effects and is the constituent herb primarily responsible for rapid antidepressant action of Yueju. It is worth noting that there were some minor differences in antidepressant response between Yueju and GJ. The time course studies indicated that the antidepressant effect of GJ appeared at 2 hours after administration, whereas Yueju started at 30 minutes. Additionally, the effective dosage of GJ (0.8 g/kg) alone was modestly higher than the proportion of GJ (0.54 g/kg) in Yueju. It is likely that some other constituent herbs in Yueju play some supporting roles by accelerating and/or enhancing GJ's antidepressant effects.

BDNF is one of the best studied neurotrophic factors and numerous studies support that the antidepressant efficacy is associated with upregulation of BDNF [23, 24]. Moreover, rapid antidepressant effect of ketamine requires BDNF upregulation in the hippocampus [7]. Although earlier study on the prefrontal cortex emphasized the dependence on mTOR signaling for ketamine's rapid antidepressant response [8], a recent report demonstrated that upregulation of BDNF in the prefrontal cortex is also required for the antidepressant response of ketamine [25]. The current study demonstrated that, from 2 hours to 24 hours after administration of GJ, BDNF protein level in the hippocampus increased significantly, in line with GJ's antidepressant responses. At 30 min after administration of GJ, BDNF level did not alter, consistent with nonsignificant antidepressant response. In contrast, there were significant BDNF and antidepressant responses at 30 min after Yueju or ketamine administration [7, 12]. These observations indicate that increase of BDNF expression in the hippocampus is temporally associated with the development of antidepressant response. A longer time required for GJ than Yueju or ketamine to increase BDNF expression and induce antidepressant effects is plausibly due to the differences in timing for bioavailability, dynamics of the metabolism, and so forth. Again, some components from other constituent herbs of Yueju may facilitate the antidepressant efficacy of GJ by accelerating BDNF expression.

As a medical plant popularly used in TCM, many effective compounds in GJ have been separated and identified. GJ contained genipin, geniposide, gardenoside, genipingentiobioside, and so forth [26, 27]. A previous study reported that geniposide contributed to antidepressant-like effects of Zhi-Zi-Hou-Pu decoction [28]. The antidepressant potential of geniposide is evidenced as it has monoamine oxidase inhibitory effect [20]. However, we did not observe the rapid antidepressant-like activity of geniposide after a single administration. Previously, it has been demonstrated that genipin exhibited antidepressant-like effect, as well as increased BDNF level in the hippocampus of chronically stressed rats using repeated administrations [21]. It is unlikely, however, genipin, as the contributor to rapid antidepressant effects, since a significant part of geniposide may have been metabolized into genipin by 24 hours after administrations, but there was no antidepressant effect with variable dosages of geniposide. Additionally, GJ has very low level of genipin. The identifications of the effective compounds are currently under investigation and some compounds extracted from the supercritical of CO_2_ may be promising candidates [29].

In Yueju formula, Yue represents GJ, as thus our study scientifically demonstrates the primary role of GJ in rapid antidepressant efficacy of Yueju. There are some limitations. First, how other constituent herbs interact with GJ in Yueju is not defined. Second, the effective compounds for GJ or Yueju remain to be determined. Nonetheless, our findings made contributions to elucidating the substrates underlying rapid antidepressant effects of Yueju. We identified GJ as the key antidepressant constituent herb of Yueju that acts rapidly. Moreover, like Yueju, GJ instantly and lastingly increased the expression of BDNF in the hippocampus, which is crucial for induction of the antidepressant effect in a fast manner. Our findings lend a support to the efficacy of GJ in quickly relieving depressive symptoms and laid a foundation for further clarifying the key underlying substrates.

## Supplementary Material

The effect dosage of the ethanol-extracted Yueju pill was 2.7g/kg, in which each of five constituent herbs contributed to 0.54g/kg. We tested whether each herb constituents of Yueju showed rapid antidepressant-like potent at this dosage. In tail suspension test carried out at 24 hours post a single administration of ethanol-extracted individual herbs, we found that none of them showed a significant effect(ANOVA, F(5,47)=0.148,p=0.979).
