# Supplementary figures and images for: Rapid Antidepressant Activity of Ethanol Extract of Gardenia jasminoides Ellis Is Associated with Upregulation of BDNF Expression in the Hippocampus
Source: Evid Based Complement Alternat Med. 2015 Mar 24;2015:761238. doi: 10.1155/2015/761238 (PMC4387974; doi:10.1155/2015/761238)

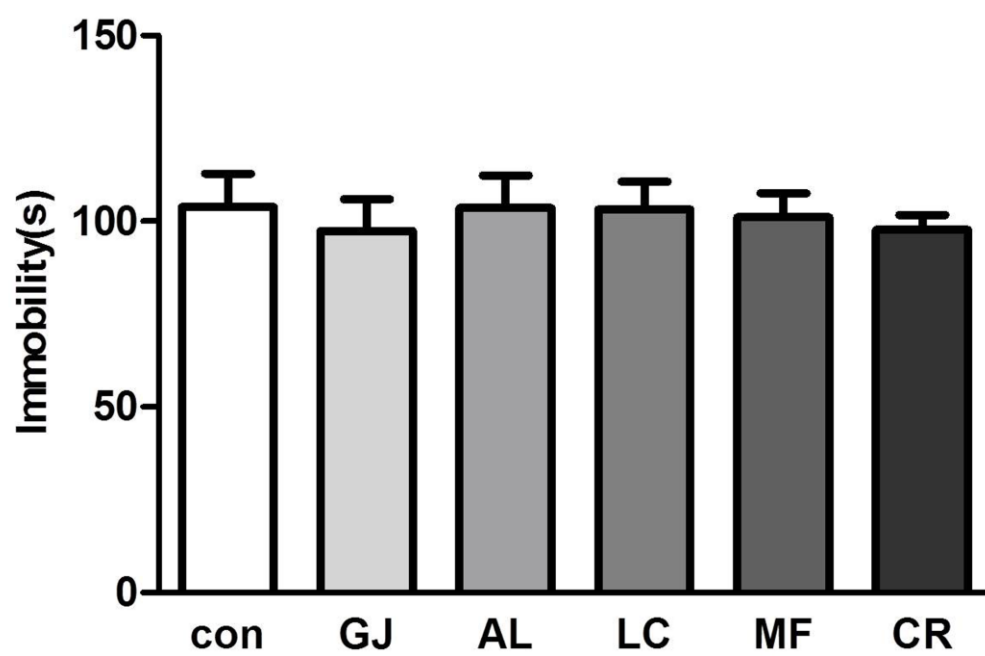

Supplement: Supplementary file 1 — The effect dosage of the ethanol-extracted Yueju pill was 2.7g/kg, in which each of five constituent herbs contributed to 0.54g/kg. We tested whether each herb constituents of Yueju showed rapid antidepressant-like potent at this dosage. In tail suspension test carried out at 24 hours post a single administration of ethanol-extracted individual herbs, we found that none of them showed a significant effect(ANOVA, F(5,47)=0.148,p=0.979). [file 761238.f1.pdf]
